# Hemispheric differences in basilar dendrites and spines of pyramidal neurons in the rat prelimbic cortex: activity- and stress-induced changes

**DOI:** 10.1111/j.1460-9568.2009.06622.x

**Published:** 2009-02

**Authors:** Claudia Perez-Cruz, Mária Simon, Boldizsár Czéh, Gabriele Flügge, Eberhard Fuchs

**Affiliations:** 1 Clinical Neurobiology Laboratory, German Primate CenterGöttingen, Germany; 2 Department of Psychiatry and Psychotherapy, Medical School, University of PécsPécs, Hungary; 3 DFG Research Center Molecular Physiology of the Brain (CMPB), University of GöttingenGöttingen, Germany; 4 Department of Neurology, Medical School, University of GöttingenGöttingen, Germany

**Keywords:** dendrite, diurnal rhythm, lateralization, prefrontal cortex, spine

## Abstract

Pyramidal neurons of the rat medial prefrontal cortex have been shown to react to chronic stress by retracting their apical dendrites and by spine loss. We extended these findings by focusing on the basilar dendritic tree of layer III pyramidal neurons in both hemispheres of the rat prelimbic cortex. Animals were subjected to daily restraint stress for 1 week (6 h/day), during either the resting or the activity period. The morphology of basilar dendrites and spines of Golgi–Cox-stained neurons in the left and right hemispheres was digitally reconstructed and analyzed. We observed the following: (i) there was an inherent hemispheric asymmetry in control rats during the resting period: the number of spines on proximal dendrites was higher in the left than in the right hemisphere; (ii) basal dendrites in controls displayed a diurnal variation: there was more dendritic material during the resting period than in the activity period; (iii) chronic stress reduced the length of basal dendrites in only the right prelimbic cortex; (iv) chronic stress reduced spine density on proximal basal dendrites; (v) restraint stress during the activity period had more pronounced effects on the physiological stress parameters than restraint stress during the resting period. Our results show dynamic hemisphere-dependent structural changes in pyramidal neurons of the rat prelimbic cortex that are tightly linked to periods of resting and activity. These morphological alterations reflect the capacity of the neurons to react to external stimuli and mirror presumptive changes in neuronal communication.

## Introduction

In humans, chronic stress-induced perturbations of the central nervous system have the potential to lead to depressive disorders (McEwen, 1998; Kendler *et al.*, 1999), and the medial prefrontal cortex (mPFC) is intimately involved in emotional processes related to such psychopathologies (Drevets *et al.*, 1997; Cardinal *et al.*, 2002; Sullivan, 2004). Studies in rats have shown that stress changes apical dendrites of pyramidal neurons in mPFC layers II–III, inducing retraction of distal dendritic branches and spine loss (Cook & Wellman, 2004; Radley *et al.*, 2004, 2006; Brown *et al.*, 2005). Repeated mild restraint stress reduces the length of apical dendrites and spine density of neurons in layer V of the mPFC (Liu & Aghajanian, 2008). However, to date no report has indicated a significant impact of stress on basal dendrites, even though the majority of synapses of neocortical pyramidal neurons are on the basal dendrites (Gordon *et al.*, 2006).

Moreover, former studies of the effect of stress on dendritic structure did not distinguish between the mPFC regions in the two hemispheres, although the reaction of the mPFC to stress is lateralized, in that responses to minor challenges stimulate the left hemisphere whereas severe stress activates the right mPFC (Sullivan, 2004). Our recent investigations indicated that hemispheric structural lateralization might exist at the cellular level in the mPFC of rats (Czéh *et al.*, 2007; Perez-Cruz *et al.*, 2007). These findings highlight the importance of analyzing the two hemispheres separately and suggest that pooling data from the two hemispheres may confound reliable effects of a treatment. Therefore, in the present study, we determined the effects of chronic stress on basilar dendrites of layer III pyramidal neurons separately in the right and the left prelimbic cortex (PL) of the rat. The PL represents the medial area of the mPFC and is an integral part of the stress circuitry (Gabbott *et al.*, 2005; Price, 2005; Vertes, 2006).

Prefrontocortical pyramidal neurons are apparently highly reactive to external stimuli, because even a daily handling procedure changed spine density on apical dendrites (Seib & Wellman, 2003). Dopamine, whose levels are elevated in the prefrontal cortex (PFC) in response to an external stimulus, is a candidate factor mediating this effect (Sullivan & Gratton, 1998). Dopamine depletion has been shown to induce spine loss in neurons of PL layer I (Wang & Deutch, 2008). However, serotonin (5-HT) and corticosterone also regulate spine number and dendritic shape (Wellman, 2001; Retana-Marquez *et al.*, 2003; see Czéh *et al.*, 2008). Common to these factors is that their brain concentrations display characteristic diurnal rhythms (Dunn *et al.*, 1972; Reul *et al.*, 1987; Feenstra *et al.*, 2000; Pérez-Vega *et al.*, 2000). For this reason, we investigated whether spine density and dendritic morphology of pyramidal neurons in the PL exhibit differences between resting and activity periods.

To expose rats to stress, we used an established experimental paradigm (Magariños & McEwen, 1995). For 1 week, male rats were daily submitted to restraint stress (6 h/day) either during the resting period or during the activity period. Subsequently, brain sections were stained using the Golgi–Cox method (Gibb & Kolb, 1998). Pyramidal neurons in layer III of the PL were three-dimensionally reconstructed and spines were counted using light microscopy as previously described (Cook & Wellman, 2004; Brown *et al.*, 2005). A Sholl (1953) analysis was performed to determine the length of basal dendrites and the number of intersections, which reflects dendritic complexity.

## Materials and methods

### Animals

Adult male Sprague–Dawley rats (Harlan-Winkelmann, Borchen, Germany) were housed in groups of three animals per cage with food and water *ad libitum* in temperature-controlled rooms (21 ± 1°C). All animal experiments were conducted in accordance with the European Communities Council Directive of November 24, 1986 (86/EEC) and the US National Institutes of Health Guide for the Care and Use of Laboratory Animals, and were approved by the Lower Saxony Federal State Office for Consumer Protection and Food Safety, Germany.

We used the minimum number of animals required to obtain consistent data. At the beginning of the habituation phase, rats weighed 150–170 g which, according to Harlan-Winkelmann, corresponds to an age of ∼6–7 weeks. To stress animals during either the resting or the activity period, one set of rats was kept on a normal light cycle (lights on at 07:00, lights off at 19:00 h), and another set was kept on an inverse light cycle (lights off at 07:00, lights on at 19:00 h). Both sets of rats contained a control and a stressed group, resulting in four experimental groups: (i) control rats on a normal light cycle; (ii) stressed rats on a normal light cycle; (iii) control rats on an inverse light cycle; and (iv) stressed rats on an inverse light cycle. Each of these four groups contained six animals. All animals were weighed daily at 08:00 h, and all handling procedures including stress exposure were performed between 08:00 and 15:00 h. In the case of the animals on the inverse light cycle, handling, weighing and restraint stress procedures were conducted under dim red light in order to avoid disruption of the light–dark cycle.

### Restraint stress

**Fig. 1:**
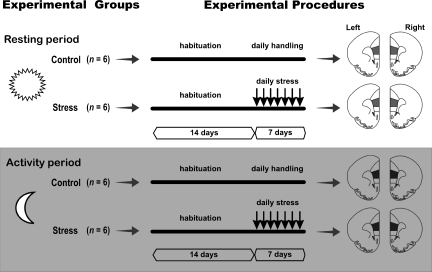
Experimental design and groups of experimental animals (for details see Materials and methods).

The experimental design is depicted in Fig. 1. The first experimental phase (habituation) lasted 14 days, during which body weight was recorded daily. During the second phase, which lasted for 7 days, animals were daily exposed to restraint stress as previously described (Brown *et al.*, 2005; McLaughlin *et al.*, 2007). Animals were immobilized daily for 6 h, from 09:00 to 15:00 h. Accordingly, for animals on the normal light cycle the restraint stress took place during their resting period (light phase). In contrast, for the animals on the inverse light cycle, the restraint stress took place during their activity period (dark phase). During restraint, each rat was placed in a plastic tube within its home cage; during this time, it had no access to food or water. Control rats were not subjected to any kind of stress except daily handling.

On day 8, in the morning, animals were again weighed, anesthetized deeply with an intraperitoneal injection of a mixture of 50 mg/mL ketamine, 10 mg/mL xylazine and 0.1 mg/mL atropine (0.2 mL/100 g of body weight), and perfused intracardially with 0.9% saline. All animals were perfused at the same time of the day, between 09:00 and 10:00 h, which corresponds to the beginning of the resting phase of the animals on the normal light cycle and to the beginning of the activity phase of the animals on the inverse light cycle. After perfusion brains were immediately dissected and processed as described below. Because increased adrenal weight is an indicator of sustained stress, adrenals were removed from the animals before perfusion and were weighed. Adrenal weight was expressed as mg/100 g of body weight.

### Golgi–Cox staining

Immediately after perfusion, the frontal part of the brain was processed for a modified Golgi–Cox staining (Gibb & Kolb, 1998). Briefly, the brains were first stored in Golgi–Cox solution in the dark for 14 days, followed by incubation in 30% sucrose in 0.85% NaCl, for 3 days. Coronal 200-μm sections were obtained at the level of the PFC using a vibratome (Vibracut 2; FTB, Bensheim, Germany); to differentiate between hemispheres the left cortex was marked with a sagittal cut. Sections were collected on clean gelatin-coated glass slides (six sections per slide), and the stain was developed with NH_4_OH for 30 min. Sections were immersed in Kodak film fixer for 30 min, washed with water, dehydrated, cleared and mounted using Eukitt (Kindler, Freiburg, Germany). The samples were covered with cover slips, and slides were dried in the dark for at least 1 month before analyzing neuronal morphology.

### Analysis of neuronal morphology

**Fig. 2:**
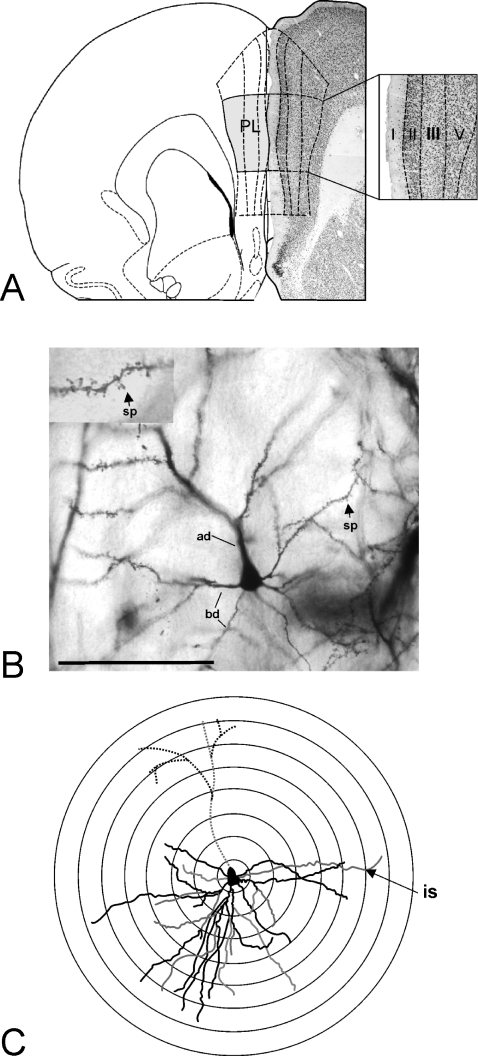
PL and Golgi–Cox-stained pyramidal neurons in layer III. (A) Representative coronal section through the prefrontal cortex (+3.7 to +2.7 mm from bregma; Paxinos & Watson, 1986) stained with NeuN antibody to visualize boundaries of the PL (see Perez-Cruz *et al.*, 2007). Dashed lines indicate boundaries of the mPFC and cortical layers, respectively. (B) Representative photomicrograph of a pyramidal neuron stained with the Golgi–Cox technique. Several basal dendrites (bd), an apical dendrite (ad) and spines (sp) can be observed. The inset shows part of a basal dendrite with spines at higher magnification. (C) Schematic illustrating reconstruction of the basal dendritic tree and Sholl circles that were used for numerical analysis. Basal dendrites are drawn as solid lines, the apical dendrite as dotted line. The points where dendrites cross the virtual circles are called intersections (is). Numbers of spines were counted in at least six dendrites proximal to soma (0–30 μm) and six dendrites distal to soma (120–200 μm). Scale bar in B, 100 μm.

Pyramidal neurons impregnated with the Golgi–Cox solution were readily identified by their characteristic, almost triangular, soma shape, apical dendrites extending toward the pial surface, and numerous dendritic spines (Robinson & Kolb, 1997). Pyramidal cells located in layer III of the PL (in both hemispheres) were identified by comparing their location in the section with a map of the PFC boundary patterns (Perez-Cruz *et al.*, 2007; Fig. 2). The criteria for digital reconstruction of neurons were: (i) soma location in PL layer III; (ii) a clear and completely stained basal dendritic tree without obviously truncated dendrites; (iii) at least three main visible basal branches, each branching at least to the third-degree branch order; and (iv) clearly visible spines (Kole *et al.*, 2004; Radley *et al.*, 2004). Cells with somata in the middle third of the section were chosen to minimize the number of truncated branches. Neurons were digitally reconstructed in three dimensions using a computer-based neuron tracing system (Neurolucida 7; MicroBrightField, Colchester, VT, USA) with the experimenter blind to the treatment condition. The morphology of the basilar arbors was then quantitatively evaluated using the Neurolucida Explorer (Version 7; MicroBrightField) as previously described (Cook & Wellman, 2004; Radley *et al.*, 2004; Brown *et al.*, 2005).

The number of neurons that were reconstructed (two or three neurons per hemisphere per animal) is depicted in Table 1. According to an established morphometric method (Cook & Wellman, 2004; Radley *et al.*, 2004; Brown *et al.*, 2005), we evaluated dendritic length and the degree of dendritic complexity using the Sholl (1953) analysis. This method estimates the amount and distribution of dendritic material at defined distances from the cell body using a virtual overlay of concentric rings centered on the soma of the neuron. In our analysis, we set the distance between the Sholl circles to 10 μm. A Sholl analysis allows determination of the length of entire dendrites and of the number of intersections. Intersections are the points where dendrites cross the virtual Sholl circles and reflect the complexity of the arbor (Fig. 2C).

**Table 1:** Number of digitally reconstructed pyramidal neurons in the PL layer III

	Control		Stress	
	Left	Right	Left	Right
Resting period	15	19	16	12
Activity period	19	18	18	19

Spine quantification was performed under a 100× objective (numerical aperture 0.75), giving a final magnification of 7000× on the monitor. Spines were counted on dendrites longer than 10 μm in at least six proximal (at a distance of 0–30 μm from the soma) and six distal (at a distance of 120–200 μm from the soma) branch segments, which represent in most cases the terminal tips of the dendrites (Fig. 2B and C). To count the spines, straight branches that provided clear resolution of spines were preferred, and spine density was calculated as the number of spines per μm of dendrite or neuron. Spines located on proximal dendrites and spines located on distal dendrites were analyzed separately.

### Statistical analysis

#### Morphometric parameters

In each hemisphere from every animal, two or three neurons were reconstructed. In a first step, the means of these two or three neurons were calculated for each respective parameter; then, these means were used to calculate the group values. Data on total dendritic length, total number of intersections and spine density were analyzed using a three-way anova. To test for group differences, the Student–Newman–Keuls method was used as *post hoc* analysis. When a more detailed Sholl analysis was conducted, i.e. when numbers of intersections or dendritic length were determined at increasing distances from soma, the areas under the curves (AUCs) were estimated and group means of the AUCs were compared using a Student’s *t*-test.

#### Physiological parameters

Body weight was measured on the last day of the experiment. Body weights and relative adrenal gland weights (mg/100 g of body weight) were analyzed using a two-way anova followed by a simple main-effect anova where appropriate.

All data are presented as group means ± SEM. The spss sigmastat 3.0 statistical software package (SigmaStat 3.0.1; SPSS Inc., Chicago, IL, USA). was used for analysis of the data. Differences were considered significant at *P*<0.05.

## Results

### Basilar dendrites of pyramidal neurons in PL layer III

We analyzed the morphology of pyramidal neurons located in layer III of the PL (Fig. 2A). Golgi–Cox-stained neurons displayed an elaborate dendritic tree with clearly distinguishable spines (Fig. 2B). Using the Sholl analysis, with concentric circles spaced 10 μm apart, we measured the total length of basal dendrites and the length of dendritic material within the concentric Sholl rings. To evaluate the complexity of the dendritic trees we determined the number of intersections, the points where dendrites cross the virtual Sholl circles (Fig. 2C). Furthermore, we counted the number of spines in the proximal and distal parts of the basal dendrites. The results of this study are based on the digital reconstruction of 136 neurons (Table 1).

### Total dendritic length

**Fig. 3:**
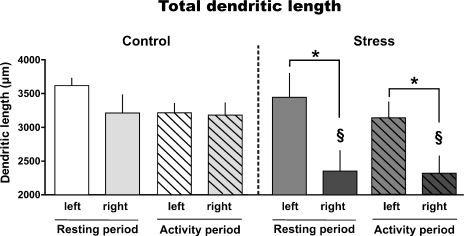
The total length of basal dendrites of pyramidal neurons in PL layer III was reduced by stress in the right hemisphere. Results of the Student–Newman–Keuls *post hoc*-test: *P<0.05 (significant difference between the hemispheres); ^§^*P*< 0.05 (compared to right hemisphere of controls in the respective period). Data are mean ± SEM.

Group values for the total length of basal dendrites in layer III pyramidal neurons are depicted in Fig. 3 and results of the statistical analysis are shown in Table 2. In control rats, no hemispheric asymmetry and no diurnal variation was detected for this parameter; however, stress reduced dendritic length of neurons in the right hemisphere. Statistical analysis with a three-way anova (stress × hemisphere × period) revealed significant main effects of stress (*F*_1,40_ = 12.68, *P*<0.001) and hemisphere (*F*_1,40_ = 16.16, *P*<0.001), and a significant interaction between stress and hemisphere (*F*_1,40_ = 8.33, *P*<0.01; Table 2). Probing of the stress ×hemisphere interaction revealed a significant main effect of hemisphere in the stress group (*q*=6.90, *P*<0.001) but not in controls (*q*=1.13, *P*=0.43). Moreover, a significant main effect of stress was detected for the right (*q*=6.45, *P*<0.001) but not for the left (*q*=0.68, *P*=0.64) hemisphere. Accordingly, stress reduced the total length of basal dendrites only in the right hemisphere (Fig. 3). The Student–Newman–Keuls test revealed that stress significantly reduced the dendritic length of neurons in the right hemisphere both in the activity and in the resting period (activity: *q*=4.00, *P*<0.05; resting: *q*=5.11, *P*<0.01, compared with the right hemisphere of controls in the same period). The stress-induced reduction in total dendritic length in the right hemisphere resulted in a hemispheric asymmetry in both activity and resting period; i.e., in stressed animals, dendrites were significantly shorter in the right hemisphere (activity period: *q*=4.33, *P*<0.05; resting period: *q*=5.43, *P*<0.01, compared with the left hemisphere in the respective period; Fig. 3).

**Table 2:** Results of the three-way anova (stress × hemisphere × period)

	Total dendritic length (Fig. 3)		Number of intersections (Fig. 5A)		Spine density (Fig. 6)	
	*F*-value	*P*-value	*F*-value	*P*-value	*F*-value	*P*-value
Main effects						
Stress	12.68	< 0.001	10.95	< 0.01	5.02	< 0.05
Hemisphere	16.16	< 0.001	8.54	< 0.01	7.59	< 0.01
Period	3.20	0.08	0.30	n.s.	13.15	< 0.01
Interactions						
Stress × hemisphere	8.33	< 0.01	14.16	< 0.001	1.02	n.s.
Stress × period	0.48	n.s.	0.28	n.s.	0.003	n.s.
Hemisphere × period	0.47	n.s.	0.04	n.s.	0.06	n.s.
Stress × hemisphere × period	0.008	n.s.	0.003	n.s.	10.10	< 0.01

n.s., not significant.

**Fig. 4:**
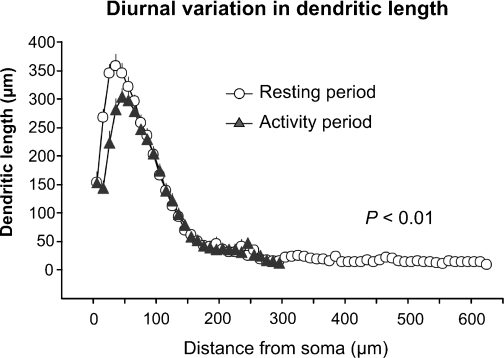
Diurnal variation in dendritic length of control rats. Comparison of basal dendritic trees of layer III pyramidal neurons revealed that, during the resting period, dendrites in the proximal segments were longer and reached further from the soma than during the activity period (for this comparison, data from the two hemispheres were pooled). Statistics: comparison of areas under the curve with Student’s *t*-test.

There was no statistically significant diurnal variation in total dendritic length (Fig. 3, Table 2). However, because a three-way anova (stress × hemisphere × period) revealed that period had a main effect close to the level of significance (*P*=0.08; Table 2, Fig. 3) we decided to carry out a more detailed Sholl analysis with the pooled data from the two hemispheres. As shown in Fig. 4, in control animals dendritic length varied greatly when expressed as a function of different distances from soma. In the resting period, dendrites reached to greater distances than in the activity period. Furthermore, in the proximal part of the dendritic tree, dendrites were more elaborate in the resting period. Comparison of areas under the curve with a Student’s *t*-test revealed a significant difference between the resting period and the activity period [*t*_(22)_ = 3.01, *P*<0.01; Fig. 4]. In the stressed animals, we could not detect a similar diurnal variation.

### Number of intersections

**Fig. 5:**
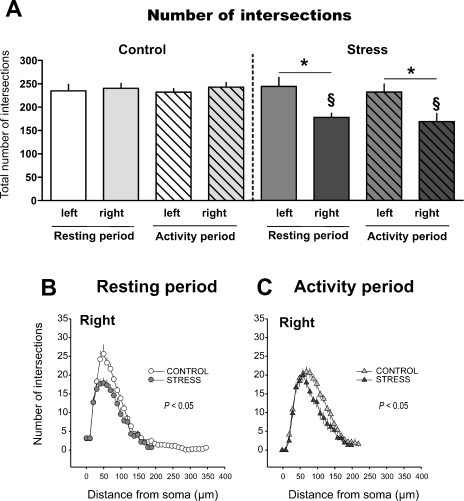
The number of intersections of basal dendrites of layer III pyramidal neurons. (A) Group values of the total number of intersections and results from three-way anova followed by Student–Newman–Keuls *post hoc*-test: *P<0.05 (difference between the hemispheres); ^§^*P*< 0.05 (difference compared to right hemisphere of controls in the respective period). (B and C) Detailed distribution of the number of intersections at increasing distances from the soma. Stress had a significant effect only in the right hemisphere, where intersections at several distances from soma were reduced. Statistics for B and C: comparison of areas under the curve with Student’s *t*-test.

The number of intersections, points where dendrites cross the virtual Sholl circles (Fig. 2C), is a parameter that reflects the complexity of the dendritic tree. Group values for the total number of intersections are depicted in Fig. 5A and results from the statistical analysis of these data are shown on Table 2. Results are similar to those of the total dendritic length, meaning that stress reduced the number of intersections in the right hemisphere. A three-way anova (stress × hemisphere × period) revealed significant main effects of stress (*F*_1,40_ = 10.95, *P*<0.01) and hemisphere (*F*_1,40_ = 8.54, *P*<0.01), and a significant interaction between stress and hemisphere (*F*_1,40_ = 14.16, *P*<0.001; Table 2). Probing of the stress × hemisphere interaction revealed that there was a hemispheric difference in the stressed animals (*q*=6.68, *P*<0.001) but not in the controls (*q*=0.84, *P*=0.56). Student–Newman–Keuls *post hoc* analysis revealed that stress significantly reduced the number of intersections in the right hemisphere in both the activity and the resting period (activity period: *q*=4.59, *P*<0.05; resting period: *q*=4.76, *P*<0.05, compared with the right hemisphere of controls during the same period). The stress-induced reduction in the total number of intersections in the right hemisphere resulted in a hemispheric asymmetry in the stressed animals, in both the activity and the resting period, i.e. the number of intersections was always lower in the right hemisphere (activity period: *q*=4.84, *P*<0.05; resting period: *q*=4.63, *P*<0.05, compared with the corresponding left hemisphere; Fig. 5A).

The graphs in Fig. 5B and C display the variation in the number of intersections at increasing distances from the soma. Stress had a significant effect only in the right hemisphere, where intersections were reduced at several distances from the soma. Comparison of areas under the curve with a Student’s *t*-test revealed a significant difference between control and stress groups both in the resting [*t*_(10)_ = 2.85, *P*<0.05; Fig. 5B] and in the activity period [*t*_(10)_ = 2.56, *P*<0.05; Fig. 5C]. In the left hemisphere, stress had no significant effect on the number of intersections (data not shown).

### Spine density

**Fig. 6:**
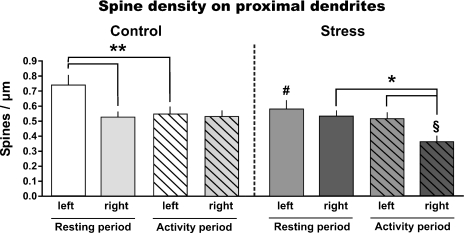
Spine density on proximal basal dendrites of pyramidal neurons in layer III. Statistical analysis of spine density with three-way anova (stress × hemisphere × activity) revealed significant main effects of all factors (see Table 2). Further group differences were analyzed with Student–Newman–Keuls *post hoc* test: *P<0.05, **P<0.01; ^§^*P*< 0.05 compared to the right hemisphere of controls in the activity period; ^#^*P* < 0.01 vs. the left hemisphere of controls during the resting period.

Group values for spine density on the proximal dendrites are depicted in Fig. 6 and the results of the statistical analysis of these data are shown in Table 2. In control rats, spine density showed a hemisphere-dependent diurnal variation and stress affected spine density in a complex manner. Statistical analysis with a three-way anova (stress × hemisphere × period) revealed significant main effects of all the three factors: stress (*F*_1,40_ = 5.02, *P*<0.05), hemisphere (*F*_1,40_ = 7.59, *P*<0.01) and period (*F*_1,40_ = 13.15, *P*<0.001; Table 2).

Moreover, analysis of group differences revealed a hemispheric asymmetry in control rats: in the resting period, spine density was ∼25% higher in the left hemisphere than in the right hemisphere (*q*=5.19, *P*<0.01). No hemispheric asymmetry was observed in the activity period (Fig. 6). The *post hoc* test also showed a diurnal variation in control animals: in the left hemisphere, spine density was higher in the resting period than in the activity period (*q*=4.95, *P*<0.01; Fig. 6).

Stress reduced spine density in either the left or the right hemisphere, depending on the period, as indicated by a significant stress × hemisphere × period interaction (*F*_1,40_ = 10.10, *P*<0.01; Table 2). In the resting period, stress reduced spine density in the left hemisphere (*q*=4.51, *P*<0.01, compared with the left hemisphere of controls in the same period). In the activity period, stress reduced spine density in the right hemisphere (*q*=3.15, *P*<0.05, compared with the right hemisphere of controls in the same period).

On the distal dendrites, no differences in spine density were detected (data not shown).

### Physiological parameters of the stress response

**Fig. 7:**
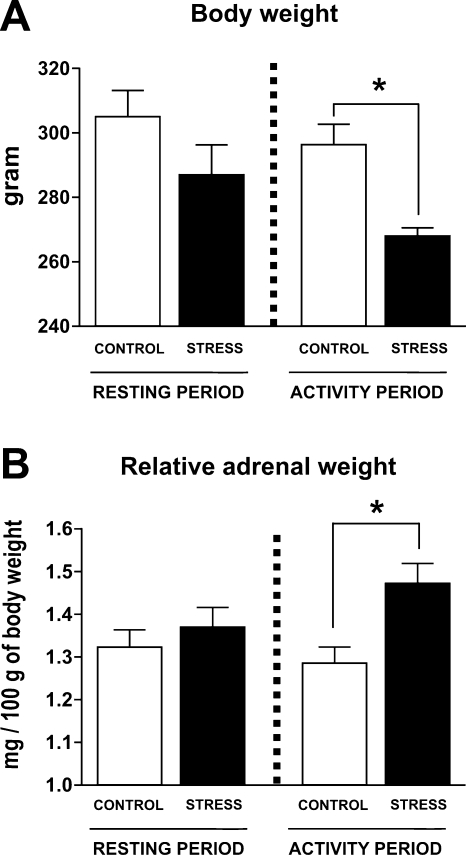
Physiological parameters showing the stress response. Note that restraint stress had a stronger impact when the animals were immobilized during their activity period. (A) Body weight (g). (B) Relative weight of the adrenal glands (mg/100 g body weight). Statistics: two-way anova followed by Student–Newman–Keuls *post hoc* test: *P < 0.05. Data are mean ± SEM.

To assess the overall physiological effects of 7 days of restraint stress, body weight and adrenal weight were determined at the end of the experiment. Stress reduced body weight in both periods, and two-way anova (stress × period) revealed a significant main effect of stress (*F*_1,20_ = 8.87, *P*<0.01). Furthermore, group comparison with the Student–Newman–Keuls *post hoc* test showed a significant difference between the control and the stress group in the activity period (*q*=3.642, *P*<0.01; Fig. 7A).

As shown previously, an increased adrenal weight after stress reflects hyperactivity of the HPA axis (Magariños & McEwen, 1995). In line with these results, we found in the present study that stress exposure increased relative adrenal weights (Fig. 7B). A two-way anova (stress × period) revealed a significant main effect of stress (*F*_1,20_ = 5.75, *P*<0.05) and a significant interaction between stress and period (*F*_1,20_ = 6.12, *P*<0.05). Student–Newman–Keuls *post hoc* analysis showed a significant difference between the control group and the stressed group in the activity period (*q* = 4.87, *P*<0.01).

## Discussion

It has been proposed that the function of the prefrontal cortex is lateralized and that the right mPFC plays a special role in integrating emotional and physiological responses to stress (Thiel & Schwarting, 2001; Sullivan, 2004). The present data demonstrate that in PL layer III, pyramidal neurons themselves show a lateralization which is modified not only by stress but also by normal time-of-day-dependent activity. We report three novel findings: (i) an intrinsic hemispheric asymmetry in the number of spines on proximal basal dendrites; (ii) a diurnal variation in the amount of dendritic material and in spine number in relation to the normal activity cycle; and (iii) a pronounced selective effect of stress on basal dendrites of neurons in the right PL.

### Lateralization and changes in relation to diurnal activity

The PFC has been implicated in higher brain functions such as selective attention, behavioral flexibility, working memory and emotional regulation, many of which show a diurnal rhythm and are lateralized (Drevets *et al.*, 1997; Groenewegen & Uylings, 2000; Thiel & Schwarting, 2001; Cardinal *et al.*, 2002). For example, studies in healthy human subjects reported on diurnal oscillations in hemisphere-dependent verbal performance and showed a decrease in the degree of left dominance from morning to evening (Corbera *et al.*, 1993). In rats, encephalograms revealed left dominance in the beginning of the resting period and right dominance when sleep pressure decreased (Vyazovskiy *et al.*, 2002). Our findings of hemispheric changes in the morphology of basal dendrites and spine number across the diurnal cycle are consistent with these studies. Morphological changes in PFC pyramidal cells might be associated with circadian oscillations in neuronal activity because dendritic geometry affects action potential propagation within a neuron with consequences for the integration of synaptic input and plasticity (Vetter *et al.*, 2001; Spruston, 2008). One may speculate that light-cycle-dependent alterations in dendritic or spine architecture of cortical pyramidal neurons contribute to the well-known diurnal changes in attention and cognitive processes (Aston-Jones & Bloom, 1981).

The present results reveal that, in control animals, dendritic architecture and spine number change within a relatively short time, i.e., from the resting to the activity period. To our knowledge, there are no previous reports addressing spine changes in the PFC across the daily cycle of resting and activity. However, rapid changes in spine density were observed in pyramidal neurons of the somatosensory cortex, where spines grow and retract within a single day (Holtmaat *et al.*, 2005). In the hippocampus of European hamsters, spines and the density of synaptic vesicles at the giant mossy fiber terminals are reduced during hibernation, but the morphology of the mossy fiber terminals found in active hamsters is restored within 2 h after arousal from torpor (Magariños *et al.*, 2006). In ground squirrels too, reduced numbers of dendritic spine enfoldings and of postsynaptic densities were observed at the mossy fiber terminals of the hippocampus in the middle of hibernation (Popov & Bocharova, 1992). *In vitro* experiments have shown that, at sites on hippocampal CA1 pyramidal neurons that show spine loss, synapse density is also reduced (Mateos *et al.*, 2007). Moreover, the morphology of pyramidal neuron spines that form synapses in layer II–III of the mouse visual cortex correlates with the size of the postsynaptic densities (Arellano *et al.*, 2007). It is therefore possible that the decrease in spines on the proximal basal dendrites of PL layer III neurons that was observed in the present study also reflects a reduced synaptic input. Proximal dendrites usually receive excitatory input from local sources or from an adjacent area (Spruston, 2008), and neurons obtain ∼90% of the excitatory input via their dendritic spines (Nimchinsky *et al.*, 2002; Spruston, 2008).

One might consider whether the observed changes in the morphology of the pyramidal neurons are related to developmental alterations. Dendrites of pyramidal neurons in the superficial layers of the mPFC in male rats reach morphological stability between 2 and 18 months of age (Grill & Riddle, 2002). In our experiment, after 2 weeks of habituation and a subsequent week of daily stress, rats were just a little older than 2 months. Therefore, it is possible that the morphology of their pyramidal neurons was still undergoing developmental changes so that the reported effects of the diurnal rhythm and of stress might relate to a particular degree of neuroplasticity within the still-developing neural network of the PL. Furthermore, it is noteworthy that we detected changes in spine density only at the proximal basal dendrites while the distal dendrites showed no significant diurnal or stress-induced alterations. These data for the distal dendrites (and their spines) do not necessarily reflect the actual situation *in vivo* because, in the 200-μm sections, some dendrites extending beyond 100 μm from soma might have been truncated. However, when selecting neurons for the digital reconstruction, we discarded all cells that displayed cut dendrites. Moreover, because of the almost horizontal orientation of most layer III pyramidal neurons within the section, many dendrites could be traced to much greater distances than 100 μm from their soma. Therefore, one can assume that truncation did not produce a systematic artifact that changed the data set significantly.

### Potential factors inducing variations in neuronal morphology

The PFC is innervated by various neurotransmitter systems that undergo diurnal changes and may modulate spine number and dendritic morphology. Circadian variations in the PFC have been described for 5-HT (Rueter & Jacobs, 1996; Nakayama, 2002), noradrenaline and dopamine, with higher levels of these monoamines registered during activity than during resting (Feenstra *et al.*, 2000; Nakayama, 2002). Serotonin changes spines and dendrites in the PL by acting on 5-HT_2A_ receptors (Miner *et al.*, 2003). There are hints that dopamine blocks dendritic growth: 1 week of daily amphetamine injections produced a long-lasting increase in the length of apical dendrites of layer III pyramidal neurons in the PFC, and it was hypothesized that this dendritic elongation was related to diminished dopaminergic input because of the chronic intervention in the dopaminergic system (Robinson & Kolb, 1997). Furthermore, release of brain-derived neurotrophic factor (BDNF) displays a circadian rhythm, with high levels during the dark phase (activity period) and low levels during the light phase (Bova *et al.*, 1998; Berchtold *et al.*, 1999). Horch *et al.* (1999) showed that overexpression of BDNF in neurons causes a dramatic loss of spines on basal dendrites.

Besides neurotransmitters and growth factors, glucocorticoids whose concentrations fluctuate during the day also induce remodeling of apical dendrites of layer II–III pyramidal neurons in the mPFC. Treating rats with corticosterone resulted in increased amounts of apical dendritic material proximal to the soma while distal apical dendrites were reduced (Wellman, 2001). Based on these findings, one may assume that numerous factors lead to morphological changes in pyramidal neurons of the PL and the distinct roles of these factors remain to be elucidated.

### Stress effects

We applied restraint stress for seven consecutive days based on a previous report showing that this type of stress causes changes in apical dendrites of layer II–III pyramidal neurons of the mPFC (Brown *et al.*, 2005). Our results demonstrate that immobilizing rats starting 2 h after beginning of the activity period had more pronounced effects on spine density than restraint starting in the beginning of the resting period. This time of day-dependent difference occurs most probably because of the distinct kind of stress: for rats, immobilization during the resting period is less stressful than immobilization during the activity period, when animals seek to move and explore their environment. Accordingly, the reduction in body weight was only reliable when rats were restrained during the activity period. Moreover, the increase in adrenal weight, which reflects hyperactivity of the HPA axis, was only significant after restraint stress during the activity period.

The effects of stress on the distinct dendritic morphological parameters appear to be mediated by different interacting factors: the reduction in spine number in the right hemisphere was only observed after immobilization during the activity period, whereas dendrites in the right hemisphere (total length and intersections) were diminished by restraint stress during both resting and activity periods. As mentioned above, there is evidence that stress effects on dendrites and spines are mediated by corticosterone (Wellman, 2001) but, apparently, other factors also play a role. One candidate factor is dopamine, whose release in the mPFC increases in response to external stimuli (Wilkinson *et al.*, 1998). Stimulus-evoked dopamine release is higher in the right than in the left PFC, which might explain why stress reduced dendrites in the right PL (Carlson *et al.*, 1993; Berridge *et al.*, 1999; Thiel & Schwarting, 2001). Besides dopamine, 5-HT (Neddens *et al.*, 2004) and noradrenaline release also increase during stress (Slopsema *et al.*, 1982). Moreover, recent findings emphasize the role of the corticotropin-releasing hormone and its receptor (CRFR1) in stress-evoked spine loss and dendritic remodeling (Chen *et al.*, 2008).

In conclusion, our data show time-of-day-dependent variations in basal dendrites of layer III pyramidal neurons in the PL and provide supporting evidence for morphological lateralization of these cortical neurons, as well as for a pronounced reduction in the length of basal dendrites of neurons in the right PL in response to stress.

## Abbreviations

5-HT: serotonin
mPFC: medial prefrontal cortex
PFC: prefrontal cortex
PL: prelimbic cortex
